# A 24-Year Analysis of Percutaneous Renal Biopsy Trends in Australia

**DOI:** 10.3390/medsci14010019

**Published:** 2025-12-31

**Authors:** Kieran Sandhu, Abdullah Al-Khanaty, Jonathon Carll, Declan G. Murphy, Nathan Lawrentschuk, Marlon Perera

**Affiliations:** 1 Division of Cancer Services, Peter MacCallum Cancer Centre, Parkville 3000, Australia; 2 Department of Urology, Royal Melbourne Hospital, Melbourne 3000, Australia; 3 Department of Surgery, Royal Melbourne Hospital, University of Melbourne, Melbourne 3000, Australia; 4 EJ Whitten Prostate Cancer Research Centre, Epworth Healthcare, Melbourne 3128, Australia; 5 Sir Peter MacCallum Department of Oncology, University of Melbourne, Melbourne 3000, Australia; 6 Department of Surgery, Austin Health, University of Melbourne, Melbourne 3084, Australia

**Keywords:** percutaneous renal biopsy, small renal masses, nephrectomy, population study

## Abstract

Background: Small renal masses (SRMs) are increasingly prevalent. Percutaneous renal biopsy (PRB) is usually reserved for diagnosis of benign renal disease or secondary metastatic disease. SRMs are frequently benign or of low malignant potential. Invasive treatment may result in significant morbidity. Current literature supports the use of PRB in diagnostic assessment of SRMs. We aim to assess the rates of PRB in Australia since its introduction in the Medicare Benefits Scheme (MBS). Methods: Data regarding PRBs for the period between January 2000 and December 2024 were extracted from the MBS. Paediatric patients aged <15 years were excluded. Population-adjusted incidences were calculated using publicly available demographic data. Results: A total of 31,870 PRBs were performed between 2000 and 2024, with an average year-on-year increase of 0.24 PRBs per 100,000 persons per year (95% CI: 0.21–0.27) and an absolute increase of 4.9 PRBs per 100,000 persons. Males had higher PRB rates than females (mean difference: 2.73 per 100,000/year). The largest rise was in NSW, increasing by 13.6 per 100,000, with similar increases in VIC and QLD (4.9 and 2.8 per 100,000, respectively). SA, TAS, and ACT experienced significant reductions (5.7, 3.5, and 6.0 per 100,000, respectively). There was significant inter-state heterogeneity (*p* < 0.001). PRB rates were highest among the 55–74 and ≥75 age groups. The PRB:nephrectomy ratio increased significantly (0.02/year; 95% CI: 0.016–0.024). Conclusions: The consensus supporting PRBs for SRMs is reflected in Australia. However, there remains significant heterogeneity between demographic groups. Further work is necessary to ensure standard-of-care treatment accessibility to prevent overtreatment.

## 1. Introduction

Small renal masses (SRMs), generally defined as lesions ≤ 4 cm, are increasing in prevalence with the increased utilisation of cross-sectional radiological imaging [1,2,3]. Percutaneous renal biopsy (PRB) has traditionally been reserved for the diagnosis of benign renal disease and renal secondary metastatic disease. SRMs are commonly benign or of low malignant potential, with rates of benign disease ranging between 19.9 and 46.3%, depending on size, and have low metastatic potential [4,5,6]. Upfront surgical or radiological intervention for SRMs may result in significant morbidity [7]. The heterogeneity of SRMs emphasises the clinical challenge of differentiating benign and malignant lesions based on imaging alone [8]. PRBs are being increasingly utilised in the evaluation of SRMs to distinguish between benign and malignant pathology and guide patient selection for surgical or radiological intervention, particularly in patients for whom surgical intervention may be high-risk due to age or the presence of co-morbidities.

Over the last two decades, there has been increasing evidence supporting the safety, diagnostic yield, and utility of PRBs. Large contemporary series have reported diagnostic yields exceeding 90% and final histopathological concordance rates exceeding 80% [9,10]. Increasingly, major urological societies have advocated for the use of PRBs in the evaluation of indeterminate renal lesions [11,12]. Despite these advances, the extent to which PRBs have been adopted in clinical practice remains poorly characterised.

Australia presents a unique setting to understand the trends in PRB uptake at a national level. Given Australia’s universal healthcare coverage, PRBs are reimbursed through the Medicare Benefits Scheme (MBS), which provides a mechanism for monitoring service delivery at a population level. Despite this universal coverage, access to specialist diagnostic procedures, including PRBs, may significantly vary depending on geographical location. Here, we aimed to characterise national trends in PRB utilisation across Australia between 2000 and 2024 and evaluated how the rates of PRB have changed over time. We specifically sought to examine whether there was significant variation by sex, age group, or state/territory. By analysing PRB rates by demographic and geographic variables, this study aims to provide insights into the longitudinal evolution and adoption of PRB in Australian clinical practice.

## 2. Methods

This was a retrospective, population-based study utilising publicly available data from the Australian MBS. National data covering the period from January 2000 to December 2024 regarding the total number of PRBs were extracted (subsidy code: 36561). MBS codes only reliably record private-practice biopsies, as private providers routinely submit claims to Medicare, while public hospitals do not. Furthermore, for MBS code 36561, there is no differentiation between PRB performed for diagnosis of medical renal disease and PRB performed for histopathological characterisation of a renal mass for surgical resection or radiologically guided ablation. Data were stratified by year, sex, age, and Australian state or territory. Australian states and territories include Victoria (VIC), New South Wales (NSW), the Australian Capital Territory (ACT), Queensland (QLD), the Northern Territory (NT), Western Australia (WA), South Australia (SA), and Tasmania (TAS). Included patients were ≥15 years of age. Patients aged <15 years were excluded to limit the analysis to adult populations where PRB is more commonly indicated and to minimize confounding from paediatric-specific renal pathology, which typically requires distinct diagnostic and management pathways [13]. Corresponding population data for 2000–2024 were extracted from the Australian Bureau of Statistics [14]. Annual service counts were converted into rates per 100,000 persons using Australian Bureau of Statistics population denominators. We modelled national and state-level trends using linear regression, with the annual index as the independent variable and the imaging rate as the dependent variable. Model estimates are reported as average change in PRBs per year with 95% confidence intervals (CIs). Linear regression was utilised, given that the MBS code does not delineate the clinical indication for PRB. Average overall and year-to-year changes were calculated nationally and for each state and expressed as both mean and median with interquartile range. Overall trends during the study period were assessed for significance. Inter-group comparisons were made using a one-way ANOVA and paired t-tests. To contextualise PRB uptake relative to surgical intervention, the annual PRB-to-nephrectomy ratio was calculated using the sum of relevant nephrectomy-related MBS codes (36522, 36525, 36526, 36527, 36528, and 36529). This ratio was evaluated over time, and linear regression was applied to assess changes in the proportion of nephrectomies to PRBs. A two-tailed *p*-value < 0.05 was considered significant. All statistical analysis was performed using GraphPad Prism version 10.5.0^®^.

## 3. Results

**Figure 1:**
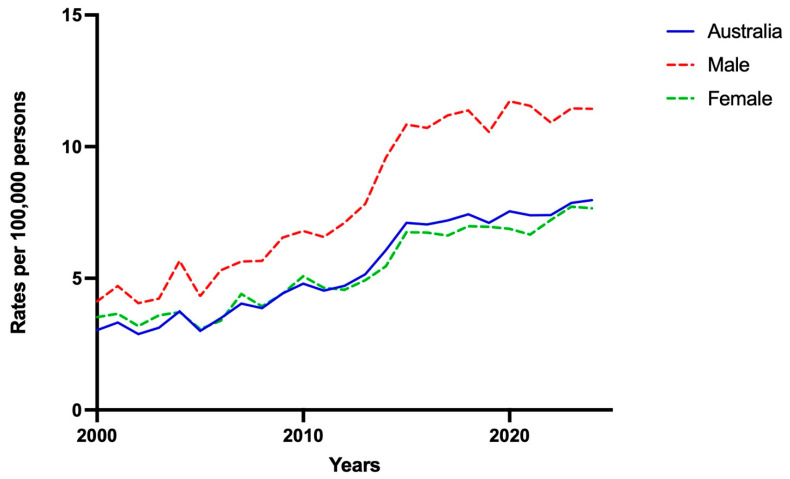
Rates of percutaneous biopsy in Australia between 2000 and 2024 with stratification by sex.

Between 2000 and 2024, a total of 31,870 PRBs were performed across Australia. Over this period, the national PRB rates more than doubled, rising from 3.03 to 7.97 per 100,000 persons between 2000 and 2024, respectively (Table 1). This represented a significant year-on-year increase of 0.24 PRBs per 100,000 (95% CI: 0.21–0.27, *p* < 0.001). The largest increase occurred between 2014 and 2015, when national rates of PRB rose by 1.03 per 100,000 persons (Figure 1). While this national growth reflects increasing adoption of PRBs overall, further analysis revealed significant disparities by sex, geographic location, and age group.

### 3.1. Sex-Based Differences

Despite overall increases in PRB use, sex-based differences were noted. PRB rates were consistently higher among males compared to females throughout the study period. In 2000, the rate among males was 4.12 per 100,000, compared to 3.53 per 100,000 in females. By 2024, this gap widened, with PRB rates of 11.44 per 100,000 in men and 7.66 per 100,000 in females. The mean difference in PRB rates between sexes was 2.73 per 100,000/year (95% CI: 1.38–4.07; *p* = 0.0002; Figure 1). Beyond sex-based differences, there was substantial variation based on geographical location.

### 3.2. Geographic Variation Across States

**Figure 2:**
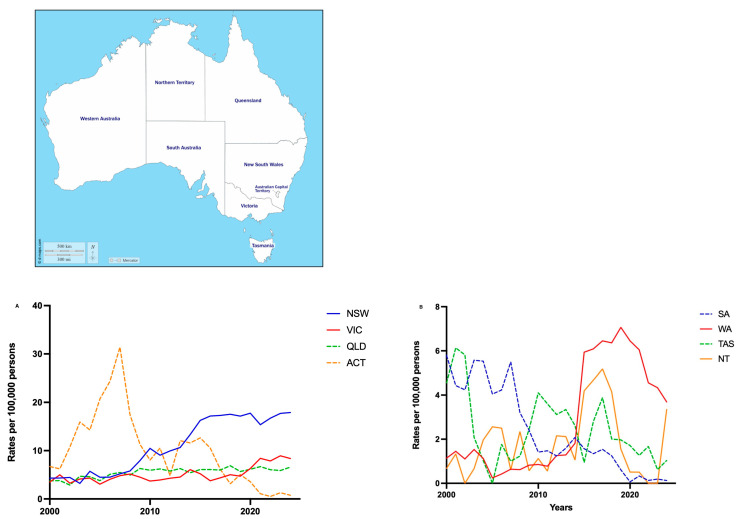
Rates of percutaneous biopsy between 2000 and 2024 stratified by state. (**A**) Eastern Australian states (NSW, VIC, QLD, and ACT); (**B**) Western and Southern Australian states (SA, WA, TAS, and NT). Map of Australia adapted from d-maps.com [https://d-maps.com/carte.php?num_car=3298&lang=en (accessed on 1 November 2025)] [15]. Rates of PRB stratified by age group.

There was marked heterogeneity in PRB adoption across Australian states (Figure 2). NSW demonstrated the largest increase, with an absolute change of 13.59 per 100,000 between 2000 and 2024, and the most rapid year-on-year increase of 0.73 per 100,000/year (95% CI: 0.62–0.83, *p* < 0.001, R^2^ = 0.90). Comparatively, several states experienced stagnation or decline in the rates of PRBs. SA and ACT showed the largest rates of reduction during the study period, with absolute decreases of −5.66 and −5.97 per 100,000, respectively. The largest difference between states occurred between NSW and SA, with a mean difference of 8.58 per 100,000 (95% CI: 5.50–11.66, *p* < 0.001). Other states, including WA (0.26 per 100,000; 95% CI: 0.15–0.37, *p* < 0.0001, R^2^ = 0.58), VIC (0.17 per 100,000, 95% CI: 0.10–0.22, *p* < 0.0001, R^2^ = 0.56), and QLD (0.11 per 100,000, 95% CI: 0.08–0.23, *p* < 0.0001, R^2^ = 0.65), demonstrated modest increases. Given the evolving role of PRBs in clinical decision-making, we also examined how utilisation differed by age group.

**Figure 3:**
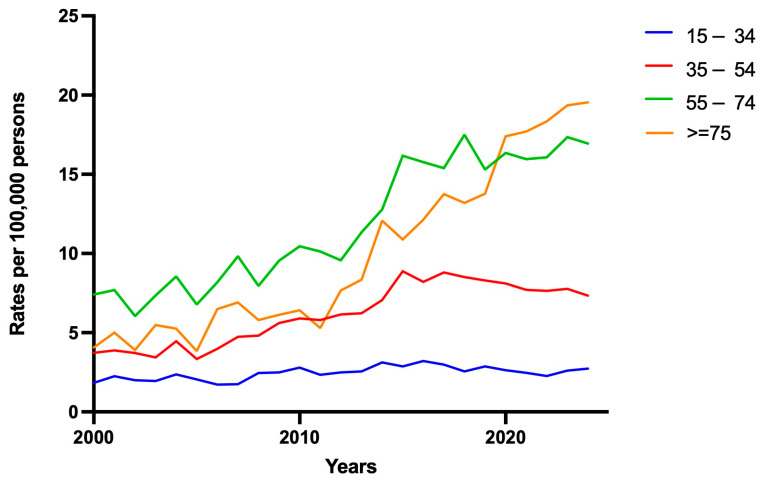
Rates of percutaneous renal biopsy between 2000 and 2024 stratified by age group.

In addition to marked sex-based and geographic variation, PRB utilisation varied significant by age group (*p* < 0.001; Figure 3). The 55–74 and ≥75-year age groups had significantly higher mean PRB rates compared to the 35–54 and 15–34 age groups, peaking at 17.50 per 100,000 in 2018 (55–74 age group) and 17.70 per 100,000 in 2021 (≥75-year age) and showing a steady year-on-year increase throughout the study period (Figure 3). A steep rise occurred in this group between 2013 and 2014 in the ≥75-year age, with a year-on-year increase of 3.73 PRBs per 100,000. The 35–54 age cohort showed moderate and stable uptake, with PRB rates increasing from 3.73–7.34 per 100,000 between 2000 and 2024, respectively. Conversely, the 15–34 age group consistently maintained the lowest rates of PRB over time, with a median of 2.49 per 100,000 (2.16–2.76), suggesting that PRBs remain uncommon in younger adults.

### 3.3. Ratio of PRBs to Nephrectomy Procedures

**Figure 4:**
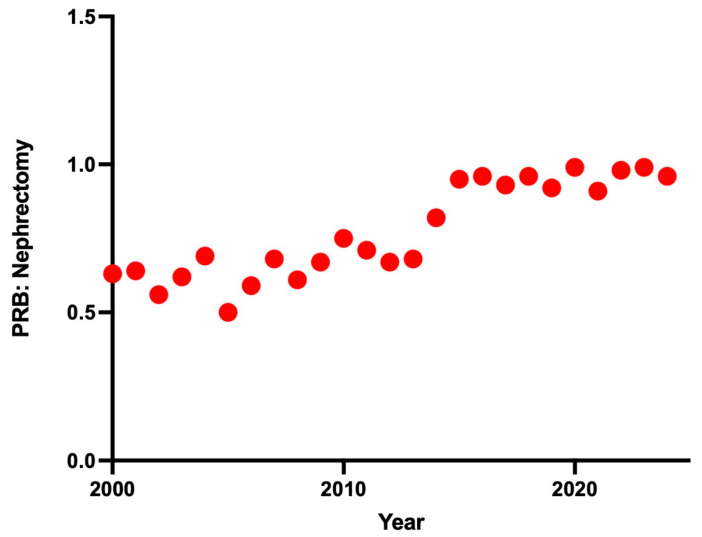
Ratio of percutaneous renal biopsies to nephrectomies in Australia between 2000 and 2024.

To account for temporal changes in renal mass detection and surgical practice, we next examined the annual ratio of PRBs to nephrectomies (including both partial and radical nephrectomies) between 2000 and 2024. Over this period, the ratio increased from 0.63 to 0.96, reflecting a significant year-on-year increase in PRB:nephrectomy ratio, with a mean annual rise of 0.02 (95% CI: 0.016–0.023, *p* < 0.0001, R^2^ = 0.81; Figure 4). The most notable rise occurred between 2013 and 2015, when the ratio rose from 0.68 to 0.95.

## 4. Discussion

This study demonstrates a significant and sustained increase in the use of PRBs in Australia over the past two decades. Between 2000 and 2024, there was an approximate doubling in the rates of PRB across Australia, with a strong linear rise on a yearly basis. These findings align with a growing international consensus supporting the safety, diagnostic utility, and expanding indications of PRBs. As PRBs are being increasingly used to distinguish benign and malignant pathology, their role in renal mass workup has shifted, becoming integral in selected patients where a nephron-sparing approach is critical. Despite an overall increase nationally, there were notable sex-based differences and marked heterogeneity between states in the utilisation of PRBs.

Sex-based differences were present throughout the study period, with males having significantly higher rates of biopsy across all years. This may be, in part, due to the underlying epidemiology of renal masses, which are more common in males, and glomerulonephritis, which is more likely to progress to end-stage renal failure in men, as well as referral biases and clinical thresholds for biopsy [16,17]. In their large, multi-centre study, Rampersaud et al. found that women aged between 42 and 58 years are more likely to present at lower stages and have a 9% reduced risk of death from renal cell carcinoma compared to men [18]. This may explain the observed lower rates of PRBs amongst women in Australia, given that PRB is often reserved for cases of indeterminate disease states.

There were notable regional differences in the adoption of PRBs. NSW maintained the highest yearly rate of PRBs and demonstrated the largest increase between 2000 and 2024. Comparatively, several states, including SA and ACT, demonstrated stagnation or even declines in the utilisation of PRBs. These differences are likely multifactorial and extend beyond access issues alone. Rural and remote regions of Australia struggle with availability of healthcare services, and the workforce in these areas often comprises early-career practitioners, leading to concerns regarding experience, specialisation, and depth of training [19]. Furthermore, the healthcare needs of people from these regions are often more complex due to higher rates of co-morbidities, which may influence the decision to provide specific interventions [20]. These issues are not specific to Australia alone, with similar patterns observed in the United Kingdom, where biopsy rates vary substantially depending on the location and local adherence to national cancer guidelines [21]. Nonetheless, delivering care locally to members of regional and rural communities not only improves access to care but aligns with patient preferences, with travel requirements for metropolitan-based care presenting significant logistical and financial burdens [22,23]. These findings emphasise the lack of equity in service provision, which may have an impact on the delivery of timely and accurate diagnosis of suspicious renal pathology. However, these regional differences may also reflect ongoing controversies regarding the utility of PRBs. Nonetheless, previous large series have demonstrated first-biopsy diagnostic yields of 90%, and histopathological concordance exceeding 80% [9,10]. Thus, the concerns surrounding PRB are largely unfounded, which is of particular importance in an ageing and co-morbid population at high risk of adverse outcomes from surgical intervention.

In addition to access-related barriers, some of the observed heterogeneity may reflect ongoing controversy surrounding the role of PRBs. Historical concerns regarding biopsy-related complications, non-diagnostic rates, and the risk of tumour seeding have likely led to some hesitation in clinical adoption, particularly among urologists. However, more recent evidence has dispelled many of these concerns, with studies showing low complication rates, negligible risk of tract seeding, and high diagnostic yields [8,9]. In the future, diagnostic yield may be enhanced by artificial intelligence approaches, but these remain under investigation [24]. Nonetheless, the legacy of this scepticism may continue to influence practice patterns unevenly across institutions and regions. Additionally, variability in training opportunities, confidence in and access to image-guided diagnostics, and a lack of standardised diagnostic pathways may contribute to heterogenous uptake [25].

Stratification by age group revealed substantial differences in PRB uptake, with the highest utilisation observed in the 55–74 and ≥75-year age brackets. These results are unsurprising, given that the prevalence of SRMs and chronic kidney disease increases with age [26]. In older patients, PRB offers the distinct advantage of enabling histological risk stratification in patients who may not be surgical candidates due to the presence of multiple co-morbidities or frailty. Furthermore, it provides an opportunity to delineate benign and malignant pathology and minimise overtreatment in a group that is more susceptible to complications. However, one recognised challenge of PRB is the difficulty in differentiating between oncocytoma and chromophobe renal cell carcinomas due to their overlapping histological and cytological features in core biopsy samples [27]. Conversely, younger adults are less likely to present with incidental renal masses in abdominal imaging and may be preferentially selected for surgery. Despite the rising rates of renal cell carcinoma in young adults, approximately 14% of lesions identified as renal cell carcinoma in imaging are found to be benign upon post-operative pathological examination [28,29]. Given the morbidity of surgical intervention and the frequent early-stage presentations in this cohort, PRBs may provide accurate diagnosis and prevent overtreatment in a young cohort, minimising the risk of renal impairment in their later adult life and providing economic advantage by mitigating unnecessary costs to the health system.

To further contextualise age-related differences, we examined the ratio of PRBs performed per nephrectomy across the study period, demonstrating a significant upward trend between 2000 and 2024. Although this may reflect enhanced uptake of image-guided diagnostics in the pre-operative workup or renal masses, it is important to acknowledge that MBS item codes do not differentiate between PRBs performed for suspected malignancy and those performed for undifferentiated medical renal disease. Thus, the rising ratio may also partially reflect increased biopsy activity in the nephrological and urological fields. Nonetheless, the upward trend reinforces the growing role of PRBs in the diagnostic landscape of renal pathology and supports efforts to integrate biopsy in routine care, particularly for patients with SRMs or those at high surgical risk.

An emerging body of evidence supports the cost-effectiveness of PRBs in the diagnostic work-up of renal masses. Modelling studies have indicated that the incorporation or PRB prior to definitive intervention reduces unnecessary surgery for benign lesions, which leads to significant cost savings and improved quality of life [30,31]. Chau et al. demonstrated that management guided by PRB findings reduced surgical intervention in approximately 12.7% of cases in their cohort study [30]. In a globally strained healthcare system, PRBs represent a rational triage tool that can enhance care and ensure that patients are appropriately selected for surgical intervention. In addition, when PRBs avert treatment in the older patient cohort, downstream costs associated with perioperative complications are likely to be reduced. These economic advantages further support the broader adoption of PRBs where appropriate.

### Limitations

There are several limitations to address in our study. Firstly, the use of MBS-based billing is dependent on accurate billing by clinicians and may not accurately capture PRBs performed outside of billing claimed via the MBS. However, MBS-based data have been validated for use in other oncological procedures, including melanoma excision [32]. Secondly, given the nature of MBS data, it is not possible to indicate the precise reason for biopsy—whether for SRM diagnosis, determining the origin of metastatic disease, or medical renal disease workup. Thirdly, age- and sex-specific denominators were derived from census estimates, which may slightly underestimate population sub-groups. Lastly, the nature of this data precludes assessment of clinical outcomes, including biopsy yield, complications, and treatment change. Nonetheless, the safety and efficacy of PRBs have been previously demonstrated [9,10].

## 5. Conclusions

This study provides a comprehensive, population-based analysis of PRB trends across Australia, revealing a significant and sustained increase in utilisation over the past two decades. While overall rates of PRB in Australia more than doubled nationally between 2000 and 2024, considerable heterogeneity was observed when rates were stratified by sex, geographical location, and age group. This heterogeneity likely reflects disparities in access to specialist services, institutional biopsy practices, and locoregional clinician attitudes. Nonetheless, these findings reflect the role of PRBs in guiding the management of SRMs in older and comorbid patients when surgical intervention may carry significant risks. Given the diagnostic and economic value of PRBs, addressing inequities in access across demographic and geographic boundaries should be prioritised.

## Figures and Tables

**Table 1:** Population and rates of percutaneous renal biopsy across Australia and its states during the study period of 2000–2024.

Location	Population in 2000	2000 Rater per 100,000	Population in 2024	2024 Rate per 100,000	Absolute Change per 100,000 Between 2000 and 2024	Mean Median (IQR)
*Australia*	19,136,268	3.03	27,380,694	7.97	+4.94	5.37 4.80 (3.75–7.20)
**States**						
*NSW*	6,484,346	4.31	8,538,848	17.9	+13.59	10.97 10.47 (5.21–17.12)
*VIC*	6,557,655	3.49	7,005,880	8.38	+4.89	5.09 4.54 (4.06–5.21)
*QLD*	3,610,810	3.74	5,617,852	6.59	+2.85	5.48 5.89 (4.92–6.15)
*SA*	1,507,587	5.79	1,890,615	0.13	−5.66	2.40 1.57 (1.24–4.23)
*WA*	1,917,553	1.14	3,008,689	3.69	+3.55	2.88 1.45 (0.86–5.94)
*TAS*	473,841	4.55	575,350	1.03	−3.52	2.43 2.01 (1.24–3.34)
*ACT*	322,855	6.72	481,235	0.75	−5.97	10.04 10.53 (4.84–12.66)
*NT*	201,731	0.675	262,225	3.35	+2.68	1.77 1.33 (0.58–2.50)

## Data Availability

The original contributions presented in this study are included in the article. Further inquiries can be directed to the corresponding author.
